# Internal rotation in the 3rd position as a candidate marker for multi-year shoulder ROM monitoring in collegiate pitchers: a 4-year individual follow-up of six clinical measures

**DOI:** 10.1186/s13102-026-01823-5

**Published:** 2026-06-24

**Authors:** Shotaro Teruya, Ryuhei Michinobu, Hiroki Yamada, Hiromitsu Tsuge, Ken Katahira, Keisei Kosaki, Kazuhiro Ikeda, Akira Ikumi, Yuki Hara, Takeshi Ogawa, Takashi Kawamura, Hiroshi Kamada, Yuichi Yoshii

**Affiliations:** 1 Department of Orthopaedic Surgery, Institute of Medicine, University of Tsukuba, 1-1-1 Tennodai, Tsukuba, Ibaraki 305-8575 Japan; 2 Department of Orthopaedic Surgery, Ichihara Hospital, 3681 Osone, Tsukuba, Ibaraki 300-3295 Japan; 3 Institute of Health and Sport Sciences, University of Tsukuba, 1-1-1 Tennodai, Tsukuba, Ibaraki 305-8574 Japan; 4 Center for Sports Medicine and Health Science, University of Tsukuba Hospital, 2-1-1 Amakubo, Tsukuba, Ibaraki 305-8576 Japan; 5 Department of Orthopaedic Surgery, National Center of Neurology and Psychiatry, 4-1-1 Ogawahigashimachi, Kodaira, Tokyo 187-8551 Japan; 6 Department of Orthopedic Surgery, NHO, Mito Medical Center, 280 Sakuranosato, Ibaraki-machi, Higashiibaraki-gun, Ibaraki 311-3193 Japan; 7 Department of Orthopaedic Surgery, Tokyo Medical University Ibaraki Medical Center, 3-20-1, Amimachi-chuo, Ami, Inashiki, Ibaraki 300-0395 Japan

**Keywords:** Baseball, Pitching, Shoulder, Range of motion, Internal rotation, Longitudinal monitoring

## Abstract

**Background:**

This study aimed to characterize four-year longitudinal changes and inter-individual variability in shoulder range of motion (ROM) in collegiate baseball pitchers, and to identify which of six commonly used clinical ROM measures shows the most consistent longitudinal change.

**Methods:**

This longitudinal observational study included 10 collegiate baseball pitchers who underwent preseason medical check-ups annually from the first academic year through the preseason of the fourth academic year. Shoulder ROM was assessed using six commonly used clinical measures of external rotation, internal rotation, and posterior shoulder tightness, and throwing–non-throwing side differences were calculated. Linear mixed models (LMM) using all four annual time points were the primary analysis; paired t-tests with Holm adjustment (Year 1 vs Years 2, 3, and 4) served as a confirmatory analysis. Robustness was assessed with three sensitivity analyses.

**Results:**

After Holm adjustment, only internal rotation in the 3rd position (IR3) showed a significant decline from Year 1 to Year 4, whereas no other ROM measure changed significantly. Mixed-model analyses across all four time points similarly indicated a significant negative longitudinal trend in IR3 (− 3.8°/year, 95% CI − 6.6 to − 1.0; *p* = 0.009), with no meaningful trends for other measures. The IR3 finding was consistent across all sensitivity analyses, and the decline was selectively driven by the throwing side, whereas the non-throwing side remained essentially unchanged. Individual trajectories varied substantially; most pitchers showed IR3 loss over time, while some remained relatively stable.

**Conclusions:**

Long-term shoulder ROM changes among collegiate pitchers showed marked inter-individual variability. IR3 was the only measure demonstrating robust deterioration across the primary, confirmatory, and sensitivity analyses, suggesting that IR3 may be a candidate marker for longitudinal monitoring of shoulder mobility during the collegiate competitive period.

**Supplementary Information:**

The online version contains supplementary material available at https://doi.org/10.1186/s13102-026-01823-5.

## Background

Overhead throwing exposes the shoulder joint to substantial and repetitive mechanical stress, particularly in baseball pitchers [1, 2]. Repeated tensile and eccentric loading applied to the posterior structures of the glenohumeral joint during pitching is thought to induce adaptive changes in posterior shoulder tissues, potentially contributing to throwing-related shoulder disorders [3].

Alterations in shoulder range of motion (ROM) are well documented in throwing athletes [4, 5]. Increased external rotation and decreased internal rotation on the throwing side, commonly referred to as glenohumeral internal rotation deficit (GIRD), have been frequently reported [3, 6, 7]. Most previous studies, however, have focused on ROM changes within a single season or across competitive levels, demonstrating short-term adaptations associated with throwing exposure [7–10]. Consequently, how shoulder ROM evolves cumulatively over multiple years of continued competitive play remains incompletely understood.

The collegiate period represents an important phase in a baseball pitcher’s development, during which pitchers may experience substantial training and competition demands, typically when skeletal maturation is near completion. These conditions suggest that chronic mechanical stress on the shoulder may accumulate during the collegiate years, yet longitudinal studies tracking shoulder ROM across the entire collegiate period remain limited. Therefore, the purpose of this study was to investigate long-term changes and inter-individual variability in shoulder ROM among collegiate baseball pitchers over a four-year period. Specifically, we aimed to (1) identify shoulder ROM measures that change longitudinally across collegiate years and (2) quantify inter-individual variability in these changes, including comparisons across measurement positions.

## Methods

### Ethics approval and consent to participate

This study was approved by the Institutional Review Board of the University of Tsukuba (approval No. 1517–5). Written informed consent was obtained from all participants prior to participation. All procedures were conducted in accordance with the Declaration of Helsinki.

### Study design

This was a longitudinal observational study examining shoulder ROM in collegiate baseball pitchers. Assessments were conducted annually during the preseason from the first academic year through the preseason of the fourth academic year. For analyses, measurement occasions were aligned by academic year relative to each participant’s enrollment (Year 1–Year 4), regardless of calendar year.

### Participants

**Fig. 1:**
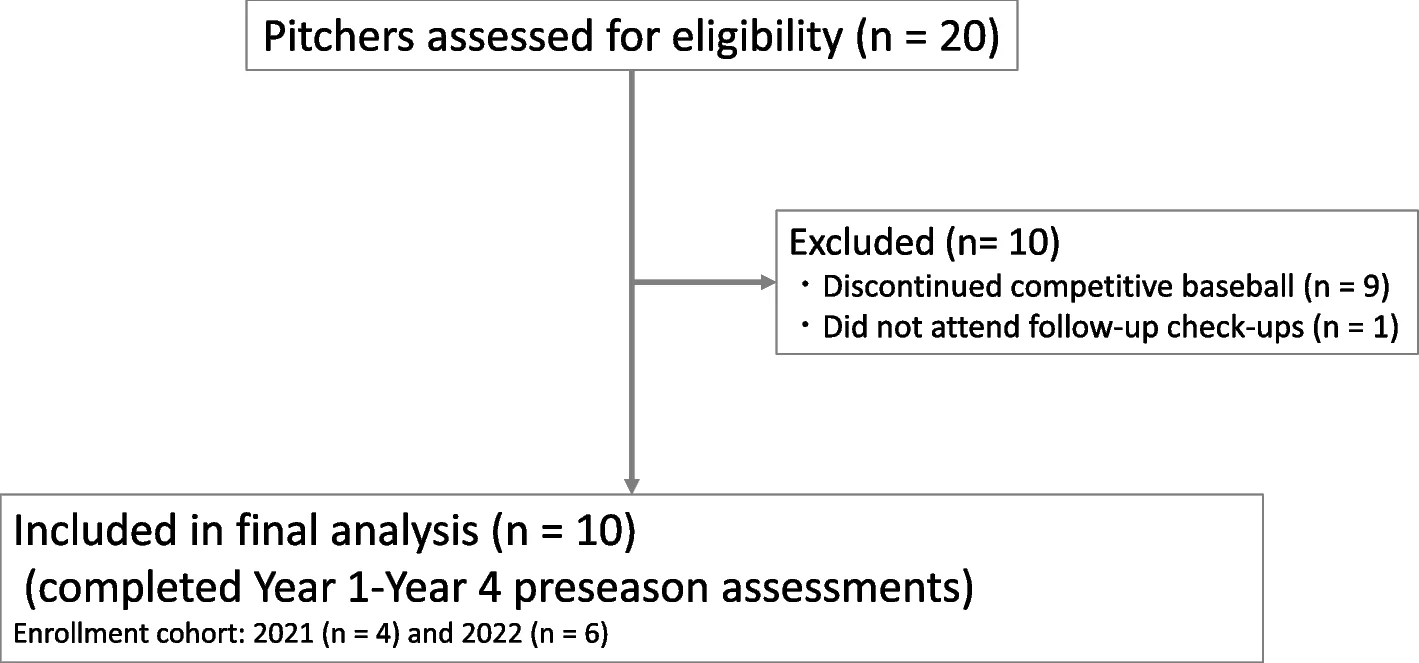
Flow of participants through the study. Twenty collegiate baseball pitchers were assessed for eligibility; 10 were excluded (discontinued competitive baseball, *n* = 9; did not attend follow-up check-ups, *n* = 1). The final analysis included 10 pitchers (2021, *n* = 4; 2022, *n* = 6) who completed all four preseason assessments (Year 1–Year 4)

Participants were pitchers from a collegiate baseball team who underwent routine annual medical check-ups. We recruited two enrollment cohorts (entered university in 2021 or 2022) and followed each participant across four preseason assessments corresponding to their first through fourth academic years. Of 20 eligible pitchers, nine discontinued competitive baseball during the study period and one did not attend follow-up check-ups; therefore, 10 pitchers (2021, *n* = 4; 2022, *n* = 6) were included in the final analysis. The demographic and clinical characteristics of the ten pitchers are summarized in Table 1. All 10 included pitchers completed assessments at all four time points with no missing data (Fig. 1).

**Table 1:** Participant characteristics (*n* = 10)

Item	Value
Age at enrollment (years)	18.3 ± 0.7 (range: 18–20)
Baseball playing career (years)	10.8 ± 1.2 (range: 9–12)
Throwing side (Right/Left)	6/4
Shoulder pain at enrollment (n)	1
Self-reported shoulder pain at any time during the 4-year follow-up (questionnaire-based, n)	6

Values are presented as mean ± SD (range) unless otherwise indicated. Categorical variables are presented as counts

### Outcome measures

Shoulder ROM was assessed using six commonly used clinical measures:

- External rotation in the abducted position (2nd position external rotation; ER2)
- External rotation in the flexed position (3rd position external rotation; ER3)
- Internal rotation in the abducted position (2nd position internal rotation; IR2)
- Internal rotation in the flexed position (3rd position internal rotation; IR3)
- Combined Abduction Test (CAT)
- Horizontal Flexion Test (HFT)

For all variables, the difference between the throwing and non-throwing sides (throwing side minus non-throwing side) was calculated and used for statistical analyses. Negative values indicate reduced ROM on the throwing side (i.e., greater restriction), whereas positive values indicate greater ROM on the throwing side. Absolute throwing-side and non-throwing-side values are also reported in Table 2 to facilitate side-specific interpretation.

**Table 2:** Descriptive statistics for shoulder ROM measures

ROM Measure	Year	Throwing side (°)	Non-throwing side (°)	Difference (°)
ER2	1	105.0 ± 15.3	98.0 ± 11.4	7.0 ± 8.2
ER2	2	117.2 ± 13.3	103.3 ± 20.5	13.0 ± 13.8
ER2	3	109.4 ± 10.2	98.1 ± 13.1	10.0 ± 12.2
ER2	4	104.0 ± 10.5	96.0 ± 15.2	8.0 ± 15.8
ER3	1	102.5 ± 9.2	100.0 ± 8.8	2.5 ± 5.4
ER3	2	106.7 ± 9.3	100.8 ± 14.6	5.0 ± 10.0
ER3	3	97.5 ± 20.4	93.3 ± 18.6	7.0 ± 9.2
ER3	4	92.0 ± 12.3	90.5 ± 8.0	1.5 ± 11.1
IR2	1	48.5 ± 16.2	58.0 ± 16.5	− 9.5 ± 15.0
IR2	2	56.7 ± 14.4	70.0 ± 18.0	− 13.0 ± 13.0
IR2	3	43.0 ± 27.7	53.0 ± 28.1	− 10.0 ± 13.1
IR2	4	33.5 ± 12.0	44.0 ± 11.5	− 10.5 ± 12.1
IR3	1	21.0 ± 17.8	21.5 ± 17.2	− 0.5 ± 5.0
IR3	2	23.0 ± 7.6	37.0 ± 14.4	− 9.5 ± 11.7
IR3	3	6.5 ± 14.9	22.5 ± 11.6	− 16.0 ± 12.9
IR3	4	10.5 ± 15.0	21.5 ± 13.8	− 11.0 ± 8.1
CAT	1	143.5 ± 14.7	141.0 ± 16.6	2.5 ± 15.7
CAT	2	156.5 ± 21.2	166.0 ± 13.1	− 9.5 ± 13.4
CAT	3	151.5 ± 7.5	163.0 ± 10.1	− 11.5 ± 8.2
CAT	4	144.5 ± 18.3	146.0 ± 19.0	− 1.5 ± 14.3
HFT	1	15.5 ± 10.7	21.5 ± 10.6	− 6.0 ± 12.2
HFT	2	21.4 ± 12.1	30.0 ± 12.9	− 7.0 ± 11.8
HFT	3	22.0 ± 5.9	33.0 ± 8.9	− 11.0 ± 8.1
HFT	4	21.5 ± 8.8	24.5 ± 8.0	− 3.0 ± 6.7

Values are mean ± SD. Throwing-side and non-throwing-side absolute values have been added; the difference values are unchanged from the original analyses. Negative difference values indicate reduced ROM on the throwing side

*ER2* external rotation in 2nd position, *ER3* external rotation in 3rd position, *IR2* internal rotation in 2nd position, *IR3* internal rotation in 3rd position, *CAT* Combined Abduction Test, *HFT* Horizontal Flexion Test

### Measurement procedures

ROM measurements were performed by trained orthopaedic physical therapists for ER and IR (10 therapists across the four-year study period) and by orthopaedic surgeons for CAT and HFT (8 surgeons across the four-year study period). Multiple examination booths operated in parallel, so the specific examiner assigned to a given participant–measure combination varied across years and across booths within the same examination day. All examiners received the same written protocol and participated in a standardized briefing immediately before each examination day, in which testing positions, anatomical landmarks, and the 0° reference convention were demonstrated and discussed. Each measurement was performed by one examiner with a second examiner independently verifying the alignment of the goniometer and the recorded value.

**Fig. 2:**
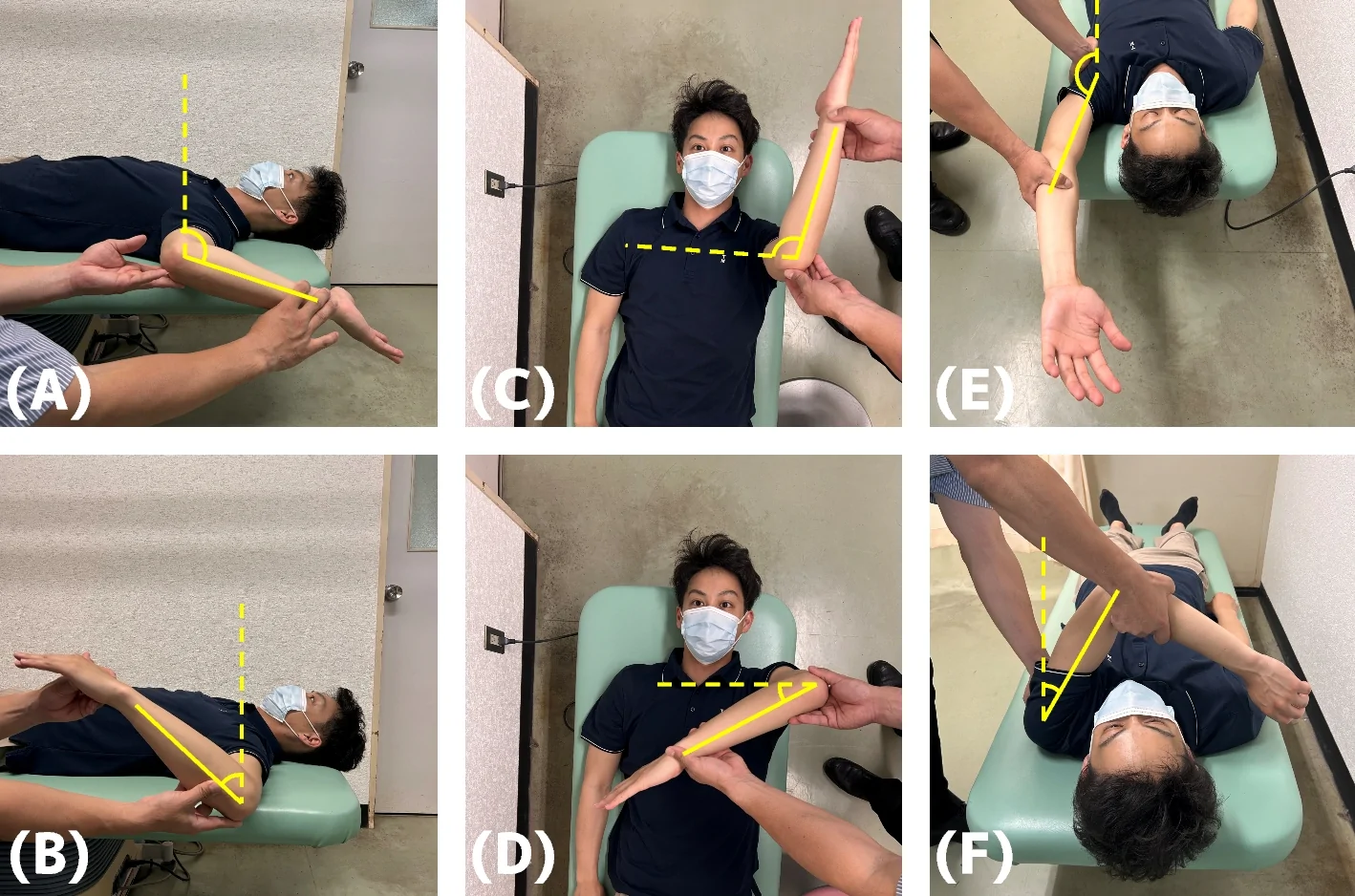
Shoulder ROM measurement positions. Photographs of the six clinical ROM measurements used in this study: **A** ER2 — external rotation in the 2nd position (supine, shoulder abducted 90°, passive); **B** IR2 — internal rotation in the 2nd position (passive); **C** ER3 — external rotation in the 3rd position (supine, shoulder flexed 90°, passive); **D** IR3 — internal rotation in the 3rd position (passive); **E** CAT — Combined Abduction Test (supine, with manual scapular stabilization, passive); **F** HFT — Horizontal Flexion Test (supine, with manual scapular stabilization, passive). The dashed yellow line in each panel indicates the 0° reference axis, and the solid yellow line indicates the measured angle. The 0° reference for the 2nd position was the forearm perpendicular to the floor; for the 3rd position, the 0° reference was the forearm parallel to the floor (oriented along the body axis)

All ROM measurements were performed using a standard goniometer as passive measurements. Each variable was measured once at each assessment session. The 2nd position was defined as 90° of shoulder abduction with 90° of elbow flexion, whereas the 3rd position was defined as 90° of shoulder flexion with 90° of elbow flexion; for both positions, ER and IR were measured in the supine position. For the 2nd position, the 0° reference was defined as the forearm perpendicular to the floor; for the 3rd position, the 0° reference was defined as the forearm parallel to the floor (oriented along the body axis). CAT was assessed in the supine position with the scapula stabilized to prevent scapular motion, evaluating glenohumeral abduction ROM only. HFT was similarly assessed in the supine position with the scapula stabilized to evaluate horizontal adduction ROM. Photographs illustrating each test position are provided in Fig. 2. To minimize measurement variability across annual assessments, all measurements followed a standardized protocol using fixed participant positioning and scapular stabilization as described above.

### Timing of assessments

Medical check-ups were conducted once during the preseason of each academic year.

### Statistical analysis

Descriptive statistics were calculated for all ROM measures at each assessment time point and are presented as mean ± standard deviation.

To leverage all four annual time points and account for individual-level variability, linear mixed models (LMM) with random intercepts for participants were used as the primary analysis (model: ROM ~ Year + (1 | Player), restricted maximum likelihood estimation). Year was modeled as a continuous variable (1–4), providing an estimate of the average annual rate of change.

To corroborate the LMM with a simpler approach using each pair of time points directly, paired t-tests were performed comparing throwing–non-throwing side differences for Year 1 vs Years 2, 3, and 4. Effect sizes were calculated using Cohen’s dz (values of 0.2, 0.5, and 0.8 considered small, medium, and large). *p*-values were adjusted using the Holm method (18 comparisons) and both unadjusted and adjusted values are reported.

Given the small sample size, three sensitivity analyses were performed to assess the robustness of the LMM result: (i) a non-parametric Friedman test with post-hoc Wilcoxon signed-rank tests; (ii) leave-one-out re-estimation of the LMM; and (iii) cluster bootstrap with player-level resampling. Detailed results are provided in Supplementary Table S1.

For ROM measures that demonstrated statistically significant deterioration in the primary or confirmatory analyses, year-by-year progression and individual longitudinal trajectories were examined to characterize inter-individual variability over the four-year period.

All analyses were performed in Python 3.12 using pandas, SciPy, and statsmodels (v0.14.0). Holm adjustment was implemented using the multipletests function from statsmodels.stats.multitest. Statistical significance was set at *p* < 0.05 for adjusted *p*-values.

## Results

### Descriptive statistics

Descriptive statistics for all ROM measures at each time point — including absolute throwing-side and non-throwing-side values — are presented in Table 2.

### Primary analysis: linear mixed models

Linear mixed model analysis revealed that among the six ROM measures (Table 3), only IR3 exhibited a statistically significant longitudinal trend. IR3 showed an average annual decline of − 3.8° per year (95% CI: − 6.6 to − 1.0; *p* = 0.009), corresponding to an estimated cumulative change of approximately − 11.4° over the four-year period. All other ROM measures showed no significant longitudinal trends in the LMM analysis (ER2: *p* = 1.000; ER3: *p* = 0.933; IR2: *p* = 1.000; CAT: *p* = 0.479; HFT: *p* = 0.724).

**Table 3:** Linear mixed model results for longitudinal ROM changes

ROM Measure	Annual Change (°/year)	95% CI	*p*-value
ER2	0.00	− 3.0, 3.0	1.000
ER3	− 0.10	− 2.4, 2.2	0.933
IR2	− 0.00	− 3.4, 3.4	1.000
IR3	− 3.80	− 6.6, − 1.0	0.009*
CAT	− 1.40	− 5.3, 2.5	0.479
HFT	0.50	− 2.3, 3.3	0.724

Linear mixed models with random intercepts for individual players (ROM ~ Year + (1|Player)). Annual change represents the estimated average rate of change per academic year. Bold text indicates statistically significant longitudinal trend (*p* < 0.05)

*CI* confidence interval, *ER2* external rotation in 2nd position, *ER3* external rotation in 3rd position, *IR2* internal rotation in 2nd position, *IR3* internal rotation in 3rd position, *CAT* Combined Abduction Test, *HFT* Horizontal Flexion Test

### Confirmatory analysis: paired comparisons

Paired t-test results comparing Year 1 with Years 2, 3, and 4 are summarized in Table 4. IR3 demonstrated a sustained decline between the first and fourth academic years, decreasing from − 0.5 ± 5.0° to − 11.0 ± 8.1° (mean change: 10.5°; 95% CI of change: 5.1 to 16.0; unadjusted *p* = 0.002; Cohen’s dz = 1.38). This finding remained statistically significant after Holm adjustment (adjusted *p* = 0.032). In the Year 1 vs Year 3 comparison, IR3 (unadjusted *p* = 0.006; adjusted *p* = 0.099) and CAT (unadjusted *p* = 0.046; adjusted *p* = 0.733) showed declines that did not survive Holm adjustment. Year 1 vs Year 2 comparisons showed no statistically significant differences for any measure after Holm adjustment. No significant differences were observed for ER2, ER3, IR2, or HFT in any comparison (all adjusted *p* > 0.05).

**Table 4:** Paired t-test comparisons (Year 1 vs Years 2, 3, and 4)

ROM measure	Comparison	Mean Change	*p*-unadj	*p*-adj	Cohen's dz
ER2	Y1 vs Y2	− 6.0	0.301	1.000	− 0.35
ER2	Y1 vs Y3	− 3.0	0.468	1.000	− 0.24
ER2	Y1 vs Y4	− 1.0	0.817	1.000	− 0.08
ER3	Y1 vs Y2	− 2.5	0.299	1.000	− 0.35
ER3	Y1 vs Y3	− 4.5	0.225	1.000	− 0.41
ER3	Y1 vs Y4	1.0	0.764	1.000	0.10
IR2	Y1 vs Y2	3.5	0.460	1.000	0.24
IR2	Y1 vs Y3	0.5	0.922	1.000	0.03
IR2	Y1 vs Y4	1.0	0.879	1.000	0.05
IR3	Y1 vs Y2	9.0	0.081	1.000	0.62
IR3	Y1 vs Y3	15.5	0.006*	0.099	1.14
IR3	Y1 vs Y4	10.5	0.002*	0.032*	1.38
CAT	Y1 vs Y2	12.0	0.094	1.000	0.59
CAT	Y1 vs Y3	14.0	0.046*	0.733	0.73
CAT	Y1 vs Y4	4.0	0.483	1.000	0.23
HFT	Y1 vs Y2	1.0	0.875	1.000	0.05
HFT	Y1 vs Y3	5.0	0.204	1.000	0.43
HFT	Y1 vs Y4	− 3.0	0.555	1.000	− 0.19

Mean change is calculated as Year 1 minus the comparison year for the throwing–non-throwing side difference (degrees). Positive values indicate increased throwing-side deficit. *p*-unadj, unadjusted *p*-value; *p*-adj, Holm-adjusted *p*-value (across 18 paired comparisons); Cohen's dz, standardized effect size for paired comparisons. Bold indicates statistical significance after Holm adjustment (adjusted *p *< 0.05). * indicates *p *< 0.05 in unadjusted analyses. Acronyms as in Table 2

### Sensitivity analyses

Detailed results of the three sensitivity analyses are provided in Supplementary Table S1. For IR3, the non-parametric Friedman test was significant (χ^2^ = 15.31, Holm-adjusted *p* = 0.010), the Year coefficient remained significant (*p* < 0.05) in 10 of 10 leave-one-out subsets, and the cluster bootstrap 95% CI for the Year 1–Year 4 mean change was [− 15.5°, − 6.0°] (excluding zero). No other ROM measure showed a significant signal in any sensitivity analysis.

### Year-by-year progression

For IR3, descriptive analysis revealed progressive deterioration from Year 1 (− 0.5 ± 5.0°) through Year 2 (− 9.5 ± 11.7°) and Year 3 (− 16.0 ± 12.9°), followed by partial improvement in Year 4 (− 11.0 ± 8.1°). Standard deviations increased substantially from Year 1 to Years 2 and 3.

Examination of the side-specific absolute values (Table 2) showed that the four-year IR3 decline was driven by the throwing side. The throwing-side IR3 decreased from 21.0 ± 17.8° at Year 1 to 10.5 ± 15.0° at Year 4, whereas the non-throwing-side IR3 remained essentially unchanged across the same period (21.5 ± 17.2° to 21.5 ± 13.8°).

For CAT, deterioration was observed from Year 1 (2.5 ± 15.7°) through Year 2 (− 9.5 ± 13.4°) and Year 3 (− 11.5 ± 8.2°), with values returning closer to baseline in Year 4 (− 1.5 ± 14.3°).

### Inter-individual variability

**Fig. 3:**
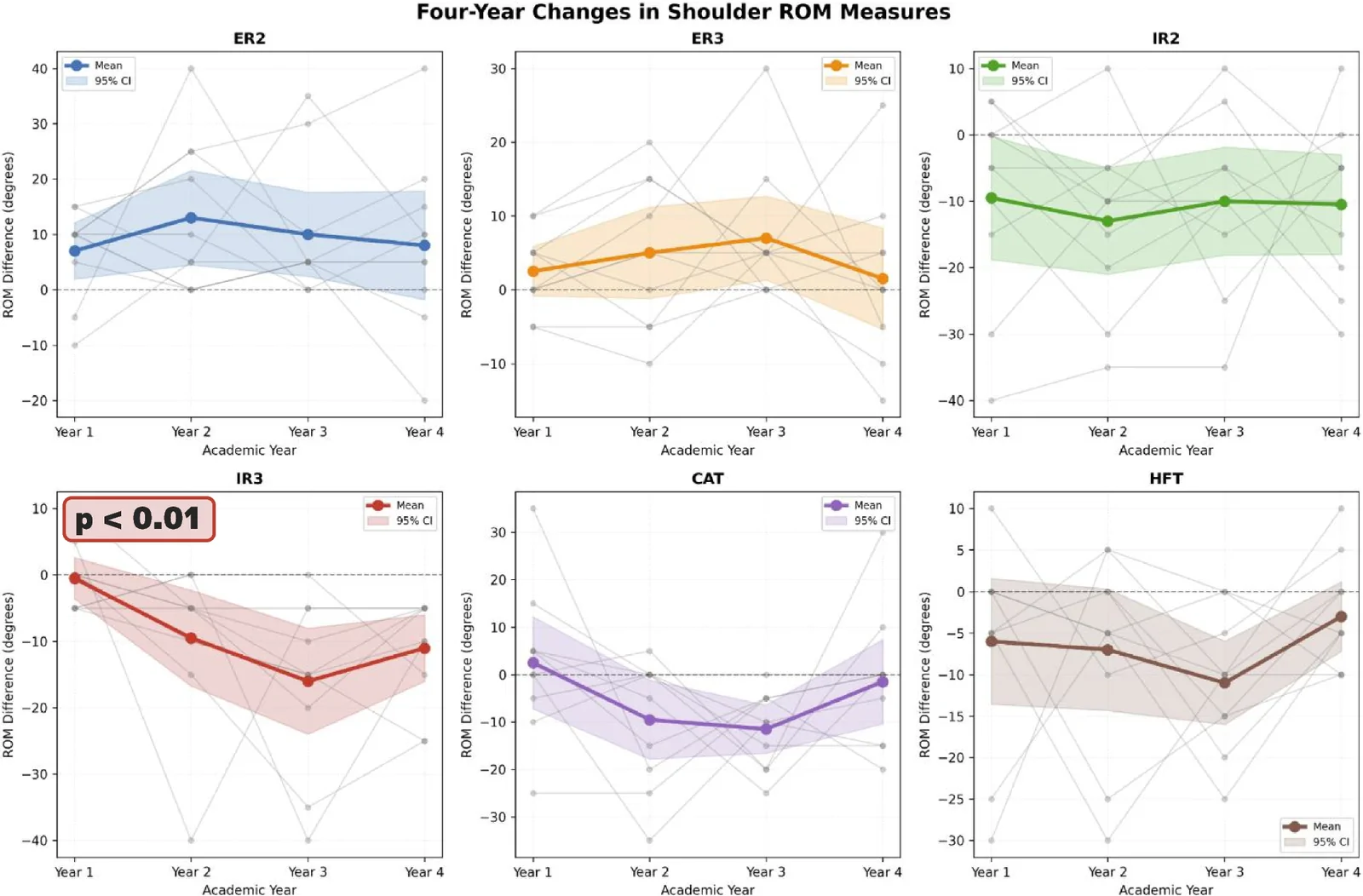
Individual trajectories and group trends for internal rotation in the 3rd position (IR3) and Combined Abduction Test (CAT) over the four-year collegiate period. Panel **A** (IR3): Individual player trajectories are shown as colored lines, classified by magnitude of change from Year 1 to Year 4: deteriorating (red, *n* = 6; change < − 5°) and stable (blue, *n* = 4; change between − 5° and 0°). The group mean is shown as a thick line with diamond markers, with shaded area representing 95% confidence interval. Linear mixed model analysis revealed a significant annual decline of − 3.8°/year (95% CI: − 6.6 to − 1.0; *p* = 0.009). Panel **B** (CAT): Individual trajectories show substantial variability with no significant group-level trend (LMM: *p* = 0.479). The dashed horizontal line in both panels indicates zero difference between throwing and non-throwing sides

Individual longitudinal trajectories for IR3 and CAT are illustrated in Fig. 3. Quantitative classification of IR3 trajectories based on Year 1 to Year 4 change revealed substantial heterogeneity: 6 pitchers (60%) demonstrated marked deterioration (change < − 5°), 4 pitchers (40%) showed relatively stable ROM (change between − 5° and 0°), and no pitcher showed improvement (positive change). The group-level LMM was significant for IR3 (*p* = 0.009) and the paired Year 1 vs Year 4 comparison was significant after Holm adjustment (adjusted *p* = 0.032). Similarly, CAT trajectories showed substantial variability, with some individuals demonstrating persistent deficits, others showing transient deterioration with subsequent recovery, and some maintaining stable or even improved values across the four-year period. However, the group-level analysis showed no significant longitudinal trend for CAT (LMM: *p* = 0.479; Year 1 vs Year 4 paired comparison: adjusted *p* = 1.000).

## Discussion

This longitudinal study investigated four-year changes and inter-individual variability in shoulder ROM among collegiate baseball pitchers. The principal findings were that (1) among six commonly used shoulder ROM measures, only internal rotation measured in the 3rd position (IR3) demonstrated significant deterioration between the first and fourth academic years and was robust across primary, confirmatory, and sensitivity analyses, and (2) longitudinal changes in IR3 were characterised by substantial inter-individual variability rather than a uniform temporal pattern. These findings underscore substantial inter-individual variability and suggest that IR3 may be useful for longitudinal monitoring in collegiate pitchers.

### Position-specific differences in sensitivity: IR3

A key finding was that only IR3 showed robust longitudinal deterioration, whereas IR2 and the other measures did not. This pattern suggests that assessment position may influence the sensitivity of detecting long-term changes. Although the 2nd position is commonly used to evaluate GIRD [11], the 3rd position (90° shoulder flexion) may better reflect changes related to posterior shoulder constraints that are less apparent in conventional assessment positions. Because posterior shoulder tightness has been linked to limitations in shoulder ROM in baseball players [12, 13], the selective decline in IR3 may reflect cumulative throwing-related changes involving posterior shoulder structures. Examination of the side-specific values further showed that the four-year IR3 decline was selectively driven by the throwing side (Year 1: 21.0 ± 17.8° → Year 4: 10.5 ± 15.0°), whereas the non-throwing side remained essentially unchanged (21.5 ± 17.2° → 21.5 ± 13.8°). This side-specific pattern is consistent with prior reports of throwing-side–specific posterior shoulder adaptations [3, 12, 14]. The concordance between the LMM (annual change) and the Year 1–Year 4 paired comparison, together with the three sensitivity analyses, supports the robustness of the IR3 finding across analytic approaches.

### Transient pattern in CAT

CAT showed a transient mid-collegiate decline in the unadjusted comparison (Year 1 to Year 3, unadjusted *p* = 0.046), with values returning toward baseline by Year 4; however, this pattern did not remain statistically significant after Holm adjustment (adjusted *p* = 0.733). The temporary nature of CAT deterioration contrasted with the more sustained changes observed in IR3. Although the mechanism is uncertain, the observed recovery is consistent with the possibility that some ROM restrictions may be modifiable, as shoulder ROM deficits and posterior shoulder tightness can improve with stretching and/or manual therapy programs in overhead athletes and baseball players [15–18]. In addition, shoulder ROM changes after pitching have been shown to be dynamic and, in some cases, partially resolve within 24 h, supporting the plausibility of reversible components of ROM restriction [10, 19].

### Inter-individual variability in long-term change

Although IR3 showed a statistically robust decline from Year 1 to Year 4, longitudinal trajectories varied substantially across individuals. Some pitchers demonstrated progressive deterioration, particularly during the middle collegiate years, whereas others maintained stable ROM or exhibited partial recovery by Year 4. This heterogeneity indicates that cumulative shoulder ROM changes during the collegiate period may not follow a uniform progression and may be influenced by factors such as pitching exposure, load management, and recovery strategies [20]. In our cohort, six of ten participants reported at least one episode of shoulder pain on the institutional questionnaire during follow-up; however, the magnitude of IR3 decline did not differ between players with and without reported pain (mean − 10.0 ± 7.1° vs. − 11.2 ± 9.5°, Mann–Whitney *p* > 0.99). Although these post-hoc comparisons are underpowered, they suggest that IR3 changes may occur even in players without symptomatic problems. Accordingly, individual ROM monitoring may be most informative when interpreted relative to each athlete’s own longitudinal baseline rather than group averages.

### Performance implications

Although shoulder ROM is often discussed in relation to pitching performance, evidence directly linking glenohumeral ROM to pitch velocity is inconsistent; in collegiate pitchers, rotational strength—but not rotational ROM—has shown significant associations with pitch velocity and pitching kinetics [21]. Nevertheless, pitchers with glenohumeral internal rotation deficit (GIRD) may exhibit altered pitching mechanics and shoulder loading, suggesting that ROM restrictions could influence performance indirectly via movement strategy [22]. From a sports-performance perspective, maintaining throwing availability across seasons is itself a key component of performance. Therefore, individualized longitudinal ROM monitoring—particularly IR3, which showed the most sensitive long-term deterioration in this cohort—may help identify athletes who require targeted interventions to maintain shoulder function and sustained competitive participation.

### Clinical implications

Internal rotation assessed in the 3rd position may be a practical and sensitive measure for longitudinal screening in collegiate pitchers, particularly when tracked repeatedly within the same athlete. Given the variability in trajectories, clinicians should prioritize within-athlete change over time rather than relying solely on cross-sectional comparisons. CAT (combined abduction test), which is performed with scapular fixation and has been used as a clinical assessment related to posterior shoulder tightness/soft-tissue restriction in overhead athletes, may be monitored as an exploratory indicator [23–25]. Because posterior shoulder tightness and related ROM deficits can improve with stretching and/or manual therapy interventions, CAT changes may reflect potentially modifiable restrictions [15–18].

### Limitations

Several limitations should be acknowledged. First, the sample size was small (*n* = 10), limiting statistical power and generalisability and the precision of variance components in the linear mixed model; therefore, these findings should be interpreted cautiously as hypothesis-generating rather than confirmatory. We addressed this concern through three sensitivity analyses (Supplementary Table S1), all of which supported the IR3 result; nevertheless, replication in larger cohorts is essential. An a priori power analysis was not feasible at the time of cohort recruitment, and a confirmatory study with a pre-specified sample size based on the IR3 effect estimate observed here is warranted. However, longitudinal studies of specific populations provide valuable samples for longitudinal monitoring of shoulder function and hypothesis generation regarding long-term changes. In addition, ROM was assessed by different examiners across years, and intra-/inter-rater reliability was not formally tested. Although all examiners followed a standardized protocol, inter-examiner variability and single-trial measurements may have introduced random error, which likely attenuated true longitudinal effects.

Second, this study describes intra-individual changes during the collegiate competitive period and did not include a control group of non-throwing athletes or non-athletes; consequently, the observed IR3 changes cannot be definitively attributed to throwing exposure rather than to age, general developmental factors, or unmeasured cohort-level influences. The side-specific pattern of decline (throwing side only) is consistent with throwing-related mechanisms, but causality cannot be established from this design.

Third, key explanatory factors and mechanisms were not fully evaluated. Quantitative pitching exposure, velocity, structured intervention adherence, and detailed in-season injury records were not systematically collected, preventing evaluation of dose–response relationships between workload and ROM changes, and players may have received interventions during the study period as part of routine care, which could have influenced individual trajectories and limits causal inference [20]. In addition, potential osseous adaptations such as humeral retrotorsion were not assessed; therefore, the mechanisms underlying changes in IR3 cannot be determined [14, 26]. Accordingly, the descriptive CAT pattern should be considered exploratory and warrants confirmation in larger cohorts with quantified workload data. This study population represents the first two cohorts of an ongoing longitudinal monitoring program at our institution; subsequent cohorts have entered follow-up, and future studies with larger samples and concurrent workload data will allow more precise estimation of the factors that the present sample size precluded.

## Conclusion

In collegiate baseball pitchers, long-term changes in shoulder ROM over a four-year period were characterised by marked inter-individual variability and position-specific differences in sensitivity. Among the six ROM measures assessed, only internal rotation measured in the 3rd position demonstrated sustained deterioration across all four academic years. Linear mixed models revealed an average annual decline of − 3.8° (*p* = 0.009), with paired comparisons confirming a cumulative change of − 10.5° (Cohen’s dz = 1.38; adjusted *p* = 0.032) from Year 1 to Year 4. No other ROM measures showed significant longitudinal changes. The selective sensitivity of the 3rd position assessment warrants further investigation in future studies incorporating direct biomechanical measures of the pitching motion. These findings underscore the importance of individualized, longitudinal monitoring of shoulder ROM in collegiate pitchers, with particular attention to the 3rd position internal rotation assessment as a potentially sensitive indicator of cumulative changes observed during the collegiate period.

## Supplementary Information

Supplementary Material 1.

## Data Availability

Anonymized data underlying the results are available from the corresponding author upon reasonable request.

## Abbreviations

ROM: Range of motion
IR: Internal rotation
ER: External rotation
IR3: Internal rotation in the 3rd position
ER3: External rotation in the 3rd position
IR2: Internal rotation in the 2nd position
ER2: External rotation in the 2nd position
CAT: Combined abduction test
HFT: Horizontal flexion test
LMM: Linear mixed model(s)
REML: Restricted maximum likelihood
CI: Confidence interval
SD: Standard deviation
GIRD: Glenohumeral internal rotation deficit
dz: Cohen’s dz
