# Supplementary material for: Internal rotation in the 3rd position as a candidate marker for multi-year shoulder ROM monitoring in collegiate pitchers: a 4-year individual follow-up of six clinical measures
Source: BMC Sports Sci Med Rehabil. 2026 Jun 24;18:402. doi: 10.1186/s13102-026-01823-5 (PMC13555891; doi:10.1186/s13102-026-01823-5)
Supplement: Supplementary file 1 [file 13102_2026_1823_MOESM1_ESM.docx]

**Supplementary Material**

*Manuscript: Internal rotation in the 3rd position as a candidate marker for multi-year shoulder ROM monitoring in collegiate pitchers: a 4-year individual follow-up of six clinical measures*

**Supplementary Table S1. Sensitivity analyses for the IR3 finding**

To assess whether the IR3 longitudinal decline observed in the primary linear mixed model (LMM) was robust to methodological choices, three sensitivity analyses were performed: (i) non-parametric Friedman test with post-hoc Wilcoxon signed-rank tests; (ii) leave-one-out (LOO) re-estimation of the LMM; and (iii) cluster bootstrap (player-level resampling). All three analyses supported the IR3 finding, and no other ROM measure showed a robust longitudinal signal in any analysis.

**A. Non-parametric tests across the four annual time points**

Friedman test was applied to each ROM measure across Years 1–4; post-hoc Wilcoxon signed-rank tests compared Year 1 vs Year 3 and Year 1 vs Year 4. Holm adjustment was applied across the 6 Friedman tests and across the 12 Wilcoxon comparisons.

**Table S1A. Friedman test (overall test across 4 time points)**

| **ROM Measure** | **χ²** | **df** | **p-value (unadjusted)** | **p-value (Holm-adjusted)** |
| --- | --- | --- | --- | --- |
| ER2 | 0.62 | 3 | 0.892 | 1.000 |
| ER3 | 1.92 | 3 | 0.589 | 1.000 |
| IR2 | 1.96 | 3 | 0.581 | 1.000 |
| **IR3** | **15.31** | **3** | **0.0016** | **0.0096** |
| CAT | 7.27 | 3 | 0.064 | 0.385 |
| HFT | 4.83 | 3 | 0.185 | 0.738 |

**Table S1B. Wilcoxon signed-rank tests (Year 1 vs Year 3, Year 1 vs Year 4)**

| **ROM Measure** | **Comparison** | **Median change (°)** | **W** | **p-value (unadjusted)** | **p-value**  **(Holm-adjusted)** |
| --- | --- | --- | --- | --- | --- |
| ER2 | Y1–Y3 | −2.5 | 13.5 | 0.330 | 1.000 |
| ER2 | Y1–Y4 | −0.0 | 21.0 | 0.937 | 1.000 |
| ER3 | Y1–Y3 | −2.5 | 6.5 | 0.144 | 1.000 |
| ER3 | Y1–Y4 | 0.0 | 11.5 | 0.612 | 1.000 |
| IR2 | Y1–Y3 | −2.5 | 20.0 | 0.846 | 1.000 |
| IR2 | Y1–Y4 | 0.0 | 17.5 | 0.642 | 1.000 |
| **IR3** | **Y1–Y3** | **−15.0** | **1.0** | **0.014** | **0.140** |
| **IR3** | **Y1–Y4** | **−10.0** | **0.5** | **0.0039** | **0.047** |
| CAT | Y1–Y3 | −12.5 | 4.0 | 0.064 | 0.578 |
| CAT | Y1–Y4 | −2.5 | 21.0 | 0.937 | 1.000 |
| HFT | Y1–Y3 | −5.0 | 13.0 | 0.296 | 1.000 |
| HFT | Y1–Y4 | 0.0 | 15.5 | 0.469 | 1.000 |

*W = Wilcoxon test statistic. Bold indicates statistical significance after Holm adjustment (p < 0.05).*

**B. Leave-one-out (LOO) sensitivity analysis**

The LMM (ROM ~ Year + (1|Player)) was refitted ten times for each ROM measure after sequentially excluding each participant. Stability of the Year coefficient and its p-value across the 10 LOO subsets is summarized below.

**Table S1C. Stability of the LMM Year coefficient under leave-one-out resampling**

| **ROM Measure** | **Full sample Year coef. (°/yr)** | **Full sample p-value** | **LOO range of coef.** | **LOO range of p-value** | **N LOO subsets with p < 0.05** |
| --- | --- | --- | --- | --- | --- |
| ER2 | 0.00 | 1.000 | −0.39, 0.39 | 0.81, 1.00 | 0/10 |
| ER3 | −0.10 | 0.933 | −0.78, 0.78 | 0.55, 1.00 | 0/10 |
| IR2 | 0.00 | 1.000 | −1.78, 1.39 | 0.43, 1.00 | 0/10 |
| **IR3** | **−3.80** | **0.009** | **−4.28, −3.17** | **0.0008, 0.030** | **10/10** |
| CAT | −1.40 | 0.479 | −2.50, −0.39 | 0.21, 0.85 | 0/10 |
| HFT | 0.50 | 0.724 | −0.06, 1.06 | 0.45, 0.97 | 0/10 |

*LOO = leave-one-out. The IR3 Year coefficient remained negative and statistically significant (p < 0.05) in all 10 LOO subsets, indicating that the longitudinal decline is not driven by any single influential observation.*

**C. Cluster bootstrap confidence intervals**

Cluster bootstrap was performed with player-level resampling. (i) For the Year 1 → Year 4 paired mean change of each ROM measure: 5,000 bootstrap resamples; the 95% percentile CI is reported. (ii) For the IR3 LMM Year coefficient: 500 cluster bootstrap resamples; the model was refitted on each resample and the 95% percentile CI is reported.

**Table S1D. Bootstrap 95% CIs for Year 1 → Year 4 paired mean change (5,000 resamples)**

| **ROM Measure** | **Observed mean change (°)** | **95% CI** |
| --- | --- | --- |
| ER2 | 1.0 | [−6.0, 9.0] |
| ER3 | −1.0 | [−6.0, 5.5] |
| IR2 | −1.0 | [−11.0, 12.5] |
| **IR3** | **−10.5** | **[−15.5, −6.0]** |
| CAT | −4.0 | [−13.5, 6.5] |
| HFT | 3.0 | [−5.0, 13.0] |

**Table S1E. Bootstrap 95% CI for the IR3 LMM Year coefficient (500 resamples)**

| **Parameter** | **Observed (°/yr)** | **Bootstrap median (°/yr)** | **95% CI** |
| --- | --- | --- | --- |
| **IR3 Year coefficient** | **−3.80** | **−3.70** | **[−5.70, −2.05]** |

**Summary**

Across all three sensitivity analyses, IR3 was the only ROM measure showing a consistent and statistically significant longitudinal decline. Specifically: (i) the non-parametric Friedman test (Holm-adjusted p = 0.010) and the post-hoc Wilcoxon signed-rank test for Year 1 vs Year 4 (Holm-adjusted p = 0.047) both confirmed the decline; (ii) leave-one-out re-estimation showed that the IR3 Year coefficient remained negative and significant in all 10 LOO subsets, with a narrow range of estimates (−4.28 to −3.17 °/year); and (iii) the cluster bootstrap 95% CI for the IR3 Year 1 → Year 4 mean change ([−15.5, −6.0]) excluded zero, and 100% of bootstrap LMM Year-coefficient estimates were negative. No other ROM measure showed any signal of robust longitudinal change in these analyses, consistent with the primary LMM and the confirmatory paired t-tests reported in the main manuscript.
